# Effects of Canthoplasty in Preventing Secondary Ocular Surface Lesions in Brachycephalic Dogs

**DOI:** 10.3390/vetsci12090889

**Published:** 2025-09-15

**Authors:** Alcyjara Rêgo Costa, Tatiane Avelar Ribeiro, Diego Marques C. Silva, José Ribamar da Silva Júnior, Tiago Barbalho Lima, Rui M. Gil da Costa, Ana I. Faustino-Rocha, Paula A. Oliveira, Ana Lúcia Abreu-Silva

**Affiliations:** 1 Veterinary Hospital, Universidade Estadual do Maranhão, 1.000, São Luís 65055-310, MA, Brazil; alcyjara.costa@hotmail.com (A.R.C.); tatianeavelar63422@gmail.com (T.A.R.); diegomarquescs@gmail.com (D.M.C.S.); 2 Departamento das Clínicas, Universidade Estadual do Maranhão, 1.000, São Luís 65055-310, MA, Brazil; aneju@gmail.com (J.R.d.S.J.); barbalho.tiago@gmail.com (T.B.L.); 3 Departamento de Patologia, Universidade Estadual do Maranhão, 1.000, São Luís 65055-310, MA, Brazil; ruioliveira@cca.uema.br; 4 Centre for the Research and Technology of Agro-Environmental and Biological Sciences (CITAB), Institute for Innovation, Capacity Building and Sustainability of Agri-Food Production (Inov4Agro), University of Trás-os-Montes and Alto Douro (UTAD), 5000-801 Vila Real, Portugal; pamo@utad.pt; 5 Department of Zootechnics, Comprehensive Health Research Center (CHRC), University of Évora, 7004-516 Évora, Portugal; 6 Department of Veterinary Sciences, University of Trás-os-Montes and Alto Douro, 5000-801 Vila Real, Portugal

**Keywords:** brachycephalic eye syndrome, canthoplasty, dog, ophthalmic surgery

## Abstract

When detected early, canthoplasty controls the clinical signs and complications associated with accentuated eye exposure. The aim of this study was to evaluate the reduction in the palpebral fissure to prevent ophthalmic lesions secondary to brachycephalic dog syndrome. A total of 64 eyes of brachycephalic dogs were studied, divided into the control group (22 eyes submitted to traditional clinical treatment) and the treated group (42 eyes submitted to canthoplasty). Tear production, tear film break-up time (TFBUT), tear crystallization time (TCT), and the occurrence of corneal ulcers were assessed prior to treatment (T0), seven days after therapy or surgery (T7), and after 30 days (T30), and reassessments were performed every three months, until month 18. It was observed that the treated animals (treated group) showed an improvement in tear production compared to the control animals (control group). In addition, the treated animals showed a significant increase in TFBUT (T9M), which was higher than the control group. The TCT evaluation scores of the treated group decreased compared to the control group (T12M), and the occurrence of corneal ulcers was significantly higher in the control group in the medium term. We concluded that early canthoplasty in brachycephalic dogs prevented the occurrence of secondary lesions, increased tear production and quality, and reduced the occurrence of ulcers, particularly in the medium term.

## Simple Summary

When detected early, canthoplasty controls the clinical signs and complications associated with accentuated eye exposure. This study evaluated the reduction in the palpebral fissure to prevent ophthalmic lesions secondary to brachycephalic dog syndrome. A total of 64 eyes of brachycephalic dogs were studied, divided into the control group (22 eyes submitted to traditional clinical treatment) and the treated group (42 eyes submitted to canthoplasty). The treated group showed an improvement in tear production compared to the control group. In addition, the animals treated showed a significant increase in TFBUT (T9M), which was higher than the control group. The TCT evaluation scores of the treated group decreased compared to the control group (T12M). The occurrence of corneal ulcers was significantly higher in the control group in the medium term. We concluded that early canthoplasty in brachycephalic dogs prevented the occurrence of secondary lesions, increased tear production and quality, and reduced the occurrence of ulcers, particularly in the medium term.

## 1. Introduction

The artificial selection of dog breeds has led to the emergence of various disorders, including ophthalmic disorders, especially in brachycephalic dogs [1]. These breeds have morphological characteristics that predispose them to lesions on the ocular surface, such as macro-blepharon, euriblepharon, shallow orbits, short muzzles with skin folds, medial entropion, hair on the caruncle, and abnormal variations [2]. These factors increase the exposure of the eyeball and make it difficult for the eyeball to close (lagophthalmos), increasing the risk of pigmentary keratitis, keratoconjunctivitis sicca, proptosis, and corneal ulcers [3]. With the growing popularity of these breeds, veterinarians and regulatory bodies are seeking to reduce these characteristics and minimize the associated disorders. Surgical reduction in the palpebral fissure is indicated for breeds such as the Lhasa Apso, Pekingese and Shih Tzu, which have exophthalmos and impaired blink reflex, leading to corneal ulcers and changes in ocular lubrication. Medial, lateral, or combined canthoplasty reduces ocular exposure, improving control of complications [4]. Many canthoplasties are performed late when lesions are already severe and vision is compromised [5]. The study’s hypothesis is that early canthoplasty may prevent damage to the ocular surface and prevent complications like dry keratoconjunctivitis and corneal ulcers. Considering the complications associated with the morphologic traits of brachycephalic breeds, combining surgical techniques to reduce the ocular surface is likely to help prevent such lesions in these canine breeds. The present study aims to assess the effects of canthoplasty with reduction in the palpebral fissure on the prevention of secondary ophthalmic lesions such as dry keratoconjunctivitis and corneal ulcers in brachycephalic dogs, and compare the results with standard clinical therapy.

## 2. Materials and Methods

The research was approved by the ethics committee (CEEA, protocol 35/2018) and carried out with the permission of the owners. Sixty-four eyes of brachycephalic dogs (6–24 months) treated at the Universidade Estadual do Maranhão (UEMA) Veterinary Hospital were analyzed. The ophthalmic examination included Schirmer’s tear test (Madhu Innovating Possibilities, New Dehli, India), a tear crystallization test (TCT) (Perfecta, São Paulo, Brazil), biomicroscopy using a portable slim lamp (Shanghai Bolan Optical Electric, Shanghai, China), tonometry using a FA-800 tonometer (Electric Suoer, St. Louis, MO, USA), ophthalmoscopy (PanOptic Plus 11820, Welch Allyn, St. Louis, MO, USA), fluorescein (Madhu Innovating Possibilities, India), tear film break-up time (TFBUT), and measurement of the palpebral fissure with a caliper (Universal 150 mm caliper, Fort G, São Paulo, Brazil). Patients were selected based on morphological characteristics such as macroblepharon and lagophthalmos, excluding those with distichiasis, ectopic eyelashes, and eyelid tumors. Physical and laboratory examinations assessed systemic conditions.

The dogs were divided into a control group, which underwent clinical treatment, and a treatment group, which underwent medial/lateral canthoplasty and entropion correction. All received topical therapy with cyclosporine, lacrimomimetics, and anti-inflammatories.

After fasting for 12 h on food and six hours on water, the treated patients (treated group) were pre-anesthetized with meperidine (0.005 mg/kg, intramuscular) and diazepam (0.3 mg/kg). Meloxicam (0.1 mg/kg, subcutaneous) was administered as a single dose. After 15 min, anesthesia was induced with propofol (5 mg/kg, intravenous) and maintained with isoflurane in a semi-closed circuit.

The surgical procedure consisted of simple medial canthoplasty [6]. The patients were positioned in the sternal decubitus position and antisepsis was performed with iodo-pyrrolidone in aqueous solution and diluted (1:50) in Ringer’s lactate. The upper and lower lacrimal canaliculi were identified and cannulated with a no. 24 catheter.

**Figure 1:**
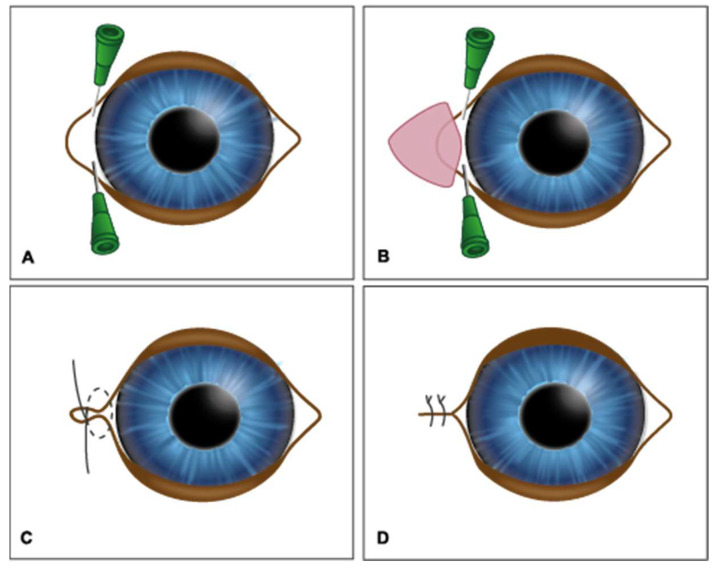
The medial canthoplasty technique. (**A**) Identification of the upper and lower lacrimal canaliculi; (**B**) resection of a skin and conjunctiva triangle, with an incision at 1–2 mm from the lacrimal canaliculi; (**C**) figure-of-eight suture to approximate and align the edges; (**D**) skin suture in a simple interrupted pattern.

A triangular section of tissue around the lateral canthus medial was carefully removed, with the base oriented towards the medial commissure and the apex directed toward the lateral commissure. The base usually measures between 2 and 4 mm. Using a no. 15 scalpel or iris scissors, the marked triangle was carefully resected, removing the skin and conjunctiva. The incision was made at a distance of 1 to 2 mm from the lacrimal canaliculi, in order to preserve those structures. To approximate and align the edges, a figure-of-eight suture was made with 5-0 nylon. The skin was then sutured in a simple interrupted pattern with 5-0 nylon, as depicted in Figure 1.

Postoperatively, the patients received meloxicam (0.1 mg/kg SID, 4 days) and tramadol (4 mg/kg, 5 days). The surgical wound was cleaned with saline solution and healing ointment, and the use of an Elizabethan collar was maintained until the stitches were removed. Evaluations took place before treatment (T0), at 7 (T7) and 30 days (T30), and every 3 months, up to 18 months (T3M-T18M), in both groups (control and treated).

Tear production was assessed by the Schirmer test, performed before any ocular manipulation, using sterile filter paper strips inserted into the conjunctival sac for one minute. The tear film break-up test (TFBUT) was carried out by instilling fluorescein into the conjunctiva, followed by manual closure of the eyelids for 10 s. The time until the first tear film rupture was timed under illumination with a cobalt blue filter, and the measurement was repeated. For the tear crystallization test (TCT), samples were collected with a microcapillary tube from the lower conjunctival sac of both eyes. The contents were placed on glass slides and analyzed under optical microscopy at 4× and 10× magnification. The classification followed the proposal by Rolando [7], categorizing the findings into four groups according to the pattern of arborization and the distance between the crystals. The presence of corneal ulcers was compared between the groups using slit lamp biomicroscopy and the fluorescein test.

The parametric data was organized in a factorial scheme (2 × 9) and submitted to analysis of variance (ANOVA), with comparison of means using the Tukey test. Qualitative variables were analyzed using the Wilcoxon test (for comparisons between groups) and the Kruskal–Wallis test (for variation over time), adopting a significance level of *p* < 0.05.

## 3. Results

A total of 64 eyes from 32 male and female brachycephalic dogs aged between 6 months and 24 months (two years) were evaluated. Of this total, 43 eyes (22 animals) that had undergone the canthoplasty procedure, the treated group, and 22 eyes (11 animals) that had undergone clinical treatment, the control group, were evaluated. One animal in the treated group had previously undergone unilateral enucleation. One animal from the treated group dropped off from T7 onwards. At T6M, one control animal suffered severe corneal perforation and underwent unilateral enucleation. The n for treated and control groups were reduced, accordingly, to 42 eyes (21 animals) and 21 eyes (11 animals), respectively.

The animals evaluated were puppies or youngsters and were already showing at least one alteration in the ocular surface associated with ocular brachycephalic syndrome, albeit discreetly.

In the evaluation of tear production, a significant increase in the Schirmer test was reported seven days after surgery in the treated group, possibly due to the surgical manipulation. After 30 days (T30), there was a reduction, followed by a progressive increase in the medium term (Table 1).

After three months (T3M) of evaluation, a progressive increase in Schirmer’s test values was reported in the animals submitted to canthoplasty, with an improvement in ocular lubrication and a reduction in mucopurulent elasticity, due to a reduction in the palpebral fissure and less exposure of the eyeball.

In the group that underwent canthoplasty (treated group), there was correction of lagophthalmos and better eyelid closure, resulting in greater protection of the ocular surface and a reduction in the consequences of keratoconjunctivitis sicca [8,9]. In the control group, there was no significant improvement in ocular lubrication, even with the use of eye drops. Furthermore, in the surgically treated group, it was possible to extend the interval of use of tear drops, reducing the long-term efficiency of canthoplasty [9]. Clinical treatment alone was not effective in all cases, requiring a surgical approach to better lubricate the ocular surface [10].

Analysis of the TFBUT showed that the animals in the treated group showed a growing increase in TFBUT values over the evaluation period (between T0 and T18M) (Table 2).

The TFBUT revealed a significant improvement in tear film quality in the surgically treated group over time, with a progressive increase in values after the procedure. However, the mean values were below what is considered normal for brachycephalic breeds (>17 s) [11]. In the control group, a reduction in mean TFBUT values was observed, reducing the worsening of tear quality in dogs submitted to clinical treatment alone (Table 2).

**Figure 2:**
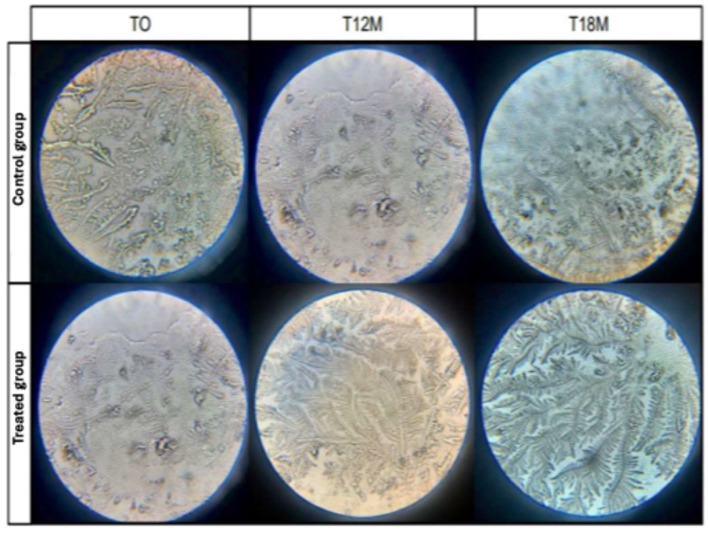
Representative images of the tear crystallization test (TCT) performed on brachycephalic canine patients before (T0) and after clinical (control group) or surgical treatment (treated group) at different times (T12M and T18M)—personal archive, 2021.

In the present study, we observed that the eyes of the animals belonging to the treated group showed a reduction in the classification scores of the TCT (Figure 2, Table 3).

In the control group, there was an increase in the TCT scores over the period evaluated, with a worsening in tear film quality, and a predominance of patterns III and IV, characteristic of dry keratoconjunctivitis [7,12]. In addition, the reduction in Schirmer’s test values coincided with the worsening of tear quality, proving quantitative and qualitative dry keratoconjunctivitis.

In the control group, even with clinical treatment, the occurrence of corneal ulcers is frequent (Table 4).

## 4. Discussion

Previous studies indicate that hereditary ophthalmic disorders in purebred dogs can appear from 6 weeks of age [13], and that puppies of brachycephalic breeds already show alterations due to ocular exposure [14]. Therefore, the inclusion of young animals was essential to evaluate preventive strategies for these lesions.

The ocular surface of brachycephalic dogs is highly susceptible to external injuries [15], and stimuli can induce greater tear production [16]. Processes caused by external agents, such as conjunctival hyperemia and edema, can rapidly develop into severe conditions [17].

Excessive ocular exposure in brachycephalic dogs is associated with ocular proliferation and the development of keratoconjunctivitis sicca [18]. Canthoplasty improves ocular lubrication by reducing corneal exposure and improving tear film distribution [19,20].

The persistence of conformational alterations in eyes treated only with topical therapy maintained poor lubrication in the central part of the cornea and could lead to subclinical inflammatory processes that impact tear production. The association of correction with clinical treatment has been shown to increase tear production in the medium term, providing better inflammatory control of the ocular surface and protective effects when performed early [21].

The TFBUT is an important diagnostic tool in the assessment of keratoconjunctivitis sicca and tear film stability in dogs [22,23]. Brachycephalic dogs are predisposed to tear film dysfunction due to exophthalmos and lagophthalmos, resulting in accelerated evaporation of the water layer [24,25]. Film rupture occurs when evaporation exceeds capillary flow, forming dark spots on the cornea [26]. Tear film instability has a direct impact on ocular health, influencing osmolarity and potentially leading to hyperosmolarity [27]. In humans, tear film osmolarity and the tear crystallization test (TCT) are used as markers of dry eye syndrome [28]. Although the TCT is used in veterinary ophthalmology [29,30], there are still no specific reports on brachycephalic breeds, and it was not measured in this study.

There was a statistically significant difference at T12M. It can therefore be inferred that there was an improvement in the quality of the tear film in the animals that underwent surgical correction in the medium and long term. According to Rolando [7] type I and II tears, which are related to a greater presence of crystals and less spacing between them, are tears of good quality, i.e., consistent with animals with a healthy ocular surface.

The TCT provides a qualitative assessment of the tear and may not be directly correlated with its quantity [31]. The interaction between electrolytes and macromolecules influences crystallization, and the absence of a halo suggests a reduction in the mucin layer and a possible decrease in goblet cells [32,33].

Ocular brachycephalic syndrome contributes to the early evaporation of the tear film and increased corneal exposure, favoring dehydration and ocular lesions [34,35]. Keratoconjunctivitis sicca can be aggravated by multiple factors, such as anatomical conformation, infections, palpebral disorders, and chronic irritations [36].

Conformational factors, such as a short snout, caruncle entropion, macrocephaly and lagophthalmos, increase the predisposition of brachycephalic dogs to corneal ulcers [3,11] observed that 81.18% of dogs with corneal ulcers were brachycephalic, reinforcing the influence of cranial morphology on their early occurrence.

Correcting anatomical abnormalities significantly reduces the risk of corneal ulcers in brachycephalic dogs [3,37]. They identified a predisposition to ulcerative keratitis, especially in young Shih Tzus (<3 years old). Recurrent ulcers occur when conformational changes persist.

Bedford & Jones [38] point out that macropalpebral fissures and shallow orbits expose the eyes of brachycephalic dogs, increasing the risk of corneal damage. Exophthalmos is also a predisposing factor for ulcerative keratitis, as it makes the ocular bulb more susceptible to trauma [37]. In addition, lagophthalmos, which is characteristic of brachycephalic ocular syndrome, compromises ocular lubrication and favors keratoconjunctivitis sicca. Eyelid and ciliary alterations, such as trichiasis and caruncle entropion, were observed in most of the animals evaluated. Caruncular trichiasis, when in constant contact with the cornea, causes chronic irritation and corneal hyperpigmentation [34,39].

Ulcerative keratitis can be associated with decreased tear production [40] or with low blink frequency and changes in tear film composition [41]. Lower corneal sensitivity in brachycephalics reduces the response to irritating stimuli and compromises tear distribution, favoring ocular lesions [42].

This study stands out among the existing literature for prospectively investigating the effects of early canthoplasty on the prevention of ophthalmic lesions in brachycephalic dogs for 18 months. Although previous studies [19] described post-surgical complications and the perception of tutors after canthoplasty, there is scant quantitative data regarding the clinical benefits obtained from this intervention [5]. Other authors [8,34] have highlighted the high prevalence of ocular lesions in brachycephalic breeds, but without exploring the impacts of an early surgical intervention. The present work contributes to filling this gap in the scientific knowledge by demonstrating that early canthoplasty performed before the development of irreversible lesions such as deep ulcers and pigmented keratitis significantly improves tear quality and reduces ocular complications. Consequently, the present results validate the efficacy of canthoplasty and support its preventive use. However, this study remains limited by its relatively low caseload, and larger studies are warranted to expand on this subject.

## 5. Conclusions

Overall, early canthoplasty in brachycephalic dogs promoted a reduction in the palpebral fissure, improved control of the quantity and quality of ocular lubrification, and prevented the mid-term effects of dry keratoconjunctivitis, supporting the study’s hypothesis. Early canthoplasty was more beneficial than conventional clinical therapy in the mid-term. Adequate follow-up remains essential to perform tear secretion control and monitor ocular health.

## Figures and Tables

**Table 1:** Schirmer’s lacrimal test performed on brachycephalic animals submitted to clinical treatment (control group) or early blepharoplasty (treated group) throughout the evaluation periods. Data are presented as mean ± standard deviation (S.D.).

Groups	Time									Mean ± S.D. Overtime
	T0	T7	T30	T3M	T6M	T9M	T12M	T15M	T18M	
Control group	14.36 ± 7.91 ^aA^	14.72 ± 7.72 ^aA^	16.6 ± 5.88 ^aA^	15.14 ± 5.47 ^aA^	14.68 ± 5.65 ^aA^	14.41 ± 4.67 ^aA^	11.00 ± 4.90 ^aA^	11.14 ± 4.83 ^aA^	11.43 ± 4.89 ^aA^	14.07 ± 6.23 ^A^
Treated group	15.74 ± 8.85 ^aA^	20.66 ± 7.05 ^bB^	17.98 ± 7.09 ^abA^	18.00 ± 5.63 ^abA^	18.22 ± 5.59 ^abB^	19.59 ± 5.71 ^bB^	20.15 ± 5.17 ^bcB^	20.86 ± 4.41 ^bcB^	23.24 ± 2.54 ^cB^	18.92 ± 6.57 ^B^

Values with equal letters, lowercase letters in the row and uppercase letters in the column, do not differ by Tukey test (*p* > 0.05). Data with normal distribution assessed by the Cramer–von Mises test at *p* > 0.05.

**Table 2:** Tear film break-up time (TFBUT) in brachycephalic animals submitted to clinical treatment (control group) or early blepharoplasty (treated group) during the evaluation periods. Data are presented as mean ± standard deviation (S.D.).

Groups	Tear Film Break-Up Time (TFBUT) (s)									Mean ±S.D. Overtime
	T0	T7	T30	T3M	T6M	T9M	T12M	T15M	T18M	
Control group	6.73 ^aA^ ± 4.32	6.91 ^aA^ ± 4.44	7.00 ^aA^ ± 3.52	6.45 ^aA^ ± 3.69	5.90 ^aA^ ± 3.66	5.27 ^aA^ ± 3.08	3.43 ^aA^ ± 1.65	3.29 ^aA^ ± 1.64	3.29 ^aA^ ± 1.64	5.64 ^A^ ± 3.63
Treated group	6.38 ^aA^ ± 3.87	6.27 ^aA^ ± 3.74	6.17 ^aA^ ± 3.48	7.32 ^abA^ ± 2.98	7.80 ^abB^ ± 3.02	9.34 ^bcB^ ± 4.26	9.81 ^cB^ ± 3.42	10.14 ^cB^ ± 2.69	11.00 ^cB^ ± 3.41	7.78 ^B^ ± 3.79

Values with equal letters, lowercase letters in the row, and uppercase letters in the column do not differ by Tukey test (*p* > 0.05). Data with normal distribution assessed by the Cramer–von Mises test at *p* > 0.05.

**Table 3:** Tear crystallization test (TCT) in brachycephalic animals submitted to clinical treatment (control group) or early blepharoplasty (treated group) during the evaluation periods. Data are presented as mean ± standard deviation (S.D.).

Groups	Tear Crystallization Test (TCT)									Mean ± S.D. Overtime
	T0	T7	T30	T3M	T6M	T9M	T12M	T15M	T18M	
Control group	2.82 ^aA^ ± 0.73	2.91 ^aA^ ± 0.68	2.91 ^aA^ ± 0.52	3.00 ^aA^ ± 0.62	3.00 ^aA^ ± 0.62	3.09 ^aA^ ± 0.52	3.29 ^aA^ ± 0.47	3.29 ^aA^ ± 0.47	3.29 ^aA^ ± 0.47	3.03 ^A^ ± 0.61
Treated group	2.90 ^aA^ ± 0.88	2.95 ^aA^ ± 0.84	2.94 ^aA^ ± 0.85	2.78 ^aA^ ± 0.69	2.78 ^aA^ ± 0.65	2.72 ^aA^ ± 0.75	2.56 ^bB^ ± 0.75	2.47 ^bB^ ± 0.75	2.29 ^bB^ ± 0.77	2.77 ^B^ ± 0.79

Values with equal letters, lowercase letters in the row and uppercase letters in the column do not differ, respectively, by the Kruskal–Wallis and Wilcoxon–Tukey tests (*p* > 0.05). Data with normal distribution assessed by the Cramer–von Mises test at *p* > 0.05.

**Table 4:** Frequency of occurrence and percentage of corneal ulcers in brachycephalic animals submitted to clinical treatment (control group) or early blepharoplasty (treated group) during the evaluation periods.

Groups	Presence or Absence of Corneal Ulcers	T7	T30	T3M	T6M	T9M	T12M	T15M	T18M
Control group	Presence	7 10.7%	0 0%	0 0%	3 4.8%	4 6.4%	3 4.8%	2 3.2%	4 6.4%
	Absent	15 23.0%	22 34.4%	22 34.4%	18 28.6%	17 27%	18 28.9%	19 30.2%	17 27%
Treated group	Present	14 21.5%	0 0%	0 0%	0 0%	0 0%	1 1.6%	1 1.6%	0 0%
	Absent	29 44.6%	42 65.65	42 65.6%	42 66.7%	42 66.7%	41 65.1%	41 65.1%	42 66.7%
Tests	Q-square (*p* value)	0.95	-	-	-	-	-	-	-
	Fisher test (*p* value)	-	1.00	1.00	0.03	0.01	0.09	0.21	0.01

Frequency of occurrence and percentage. Controls are 22 up to T3M and 21 from then onwards. Treated are 43 at T7 and 42 from then onwards. When observed frequency values are less than 5, use Fisher’s test; for values greater than 5, use the Q-squared test. Frequencies are different when *p* < 0.05.

## Data Availability

Data available under request.

## Abbreviations

The following abbreviations are used in this manuscript:

TCT	Tear crystallization test
TFBUT	Tear film break-up time
